# Associations between Adipokines in Arthritic Disease and Implications for Obesity

**DOI:** 10.3390/ijms20061505

**Published:** 2019-03-26

**Authors:** Iona J. MᵃᶜDonald, Shan-Chi Liu, Chien-Chung Huang, Shu-Jui Kuo, Chun-Hao Tsai, Chih-Hsin Tang

**Affiliations:** 1 Graduate Institute of Basic Medical Science, China Medical University, Taichung 40402, Taiwan; ionamac@gmail.com (I.J.M.); sdsaw.tw@yahoo.com.tw (S.-C.L.); 2 Department of Orthopedic Surgery, China Medical University Hospital, Taichung 40447, Taiwan; b90401073@gmail.com (S.-J.K.); ritsai8615@gmail.com (C.-H.T.); 3 Graduate Institute of Clinical Medical Science, China Medical University, Taichung 40447, Taiwan; u104054003@cmu.edu.tw; 4 Division of Immunology and Rheumatology, Department of Internal Medicine, China Medical University Hospital, Taichung 40447, Taiwan; 5 Department of Anatomy, China Medical University, Taichung 40402, Taiwan; 6 School of Medicine, China Medical University, Taichung 40402, Taiwan; 7 Chinese Medicine Research Center, China Medical University, Taichung 40447, Taiwan; 8 Department of Biotechnology, College of Health Science, Asia University, Taichung 41354, Taiwan

**Keywords:** rheumatoid arthritis, osteoarthritis, adipokines, obesity

## Abstract

Secretion from adipose tissue of adipokines or adipocytokines, comprising of bioactive peptides or proteins, immune molecules and inflammatory mediators, exert critical roles in inflammatory arthritis and obesity. This review considers the evidence generated over the last decade regarding the effects of several adipokines including leptin, adiponectin, visfatin, resistin, chemerin and apelin, in cartilage and bone homeostasis in the pathogenesis of rheumatoid arthritis and osteoarthritis, which has important implications for obesity.

## 1. Introduction

**Figure 1:**
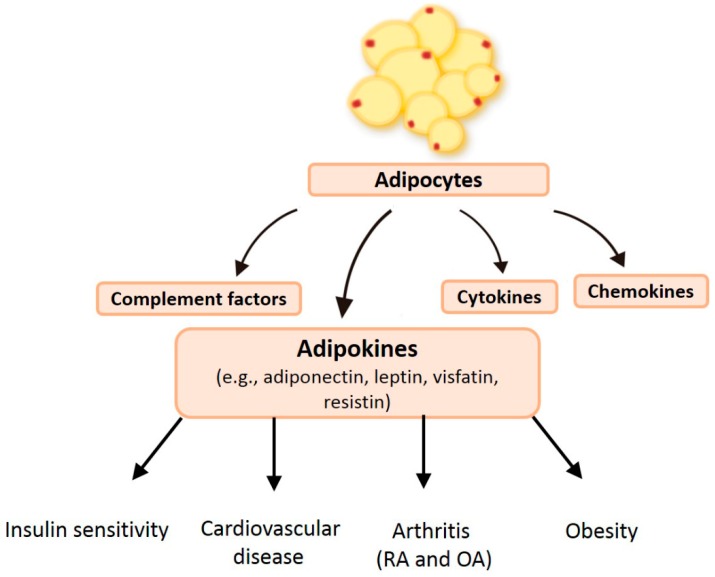
The important role of adipokines. Adipokines are produced mainly by adipocytes and play critical roles in several major disorders including insulin sensitivity, cardiovascular disease, arthritic conditions (i.e., RA and OA), and obesity.

Adipose tissue secretes various bioactive peptides or proteins, immune molecules and inflammatory mediators known as adipokines (only produced by the adipose tissue) or adipocytokines (mainly, but not solely, produced by adipocytes) (Figure 1). In this review, the term “adipokine” refers to these multifunctional molecules. Since the discovery in 1994 of the first adipokine, leptin, profiling studies have identified hundreds of adipokines in the human adipose proteome (adipokinome), all of which can potently modulate inflammation via autocrine/paracrine and endocrine pathways. Some of these multifunctional molecules are critical to the pathogenesis of rheumatoid arthritis (RA) and osteoarthritis (OA), modulating target tissues and cells in cartilage, synovium, bone, and various immune cells [1]. Thus, our review of data details adipose tissue paracrine signaling in RA and OA and discusses correlations identified between adipokines, obesity and the development of RA and OA. These are two of the most important and common arthritic diseases that lead to bone destruction and deformity; we therefore focused on the role of adipokines in these arthritic diseases. Our evidence is drawn from the period of January 2007 through October 2018, because the literature begins to extensively cover the role of adipose tissue and adipokines in obesity, RA and OA from 2007 and our literature search ended in October 2018. 

Rheumatoid arthritis, a chronic autoimmune disease marked by persistent synovial and systemic inflammation, damages joints, results in disability and increases cardiovascular burden. The pathogenesis of RA is uncertain, but the underlying pathology appears to commence outside the joints [2]. Obesity is accompanied by low-grade inflammation and is a recognized risk factor for several well-known health problems, including cardiovascular disorders, disorders of metabolic syndrome (MetS), various cancers, and some rheumatic diseases [3]. Evidence demonstrates that obesity independently increases the risk of RA developing in “at-risk”, autoantibody-positive people, and that higher birth weight is associated with the future onset of RA [4,5]. Conversely, other evidence suggests that obesity has no influence over the likelihood of developing RA. For instance, researchers have reported that in early RA, higher body mass index (BMI) not only does not influence the progression to clinical RA, but that it may be associated with less radiographic joint damage, with people who are obese developing fewer joint erosions and experiencing slower structural progression [6,7]. Interestingly, an association between lower BMI and progression of radiographic joint damage in early RA has been observed only in seropositive individuals [6,8,9].

In comparison to RA, a more definite link is established between higher BMI and the risk of developing hip and knee OA in men and women [10,11]. Evidence from the Framingham Heart Study reveals a 1.5- to 2-fold higher risk of developing knee OA among people who are obese compared with those who are leaner [1] and, in a US population-based study involving community-dwelling older adults (aged ≥70 years), a 5 kg/m^2^ increase in BMI increased the likelihood of developing knee OA by 32% [12]. Not only did a 200 pM increase in serum leptin increase the odds of knee OA by 11%, but also, approximately half of the BMI’s total effect on knee OA was attributed to leptin. In pooled relative risks (RRs) of a recent meta-analysis, overweight and obesity significantly increased the risk of knee OA by approximately 2.5 and 4.6 times, respectively, compared with normal weight [13]. Other risk factors that predispose to OA include joint trauma, and family history or medical disorders presenting with joint inflammation, such as hemochromatosis, septic arthritis, inflammatory arthritis, avascular necrosis, hemophilia, or gout [12].

As higher BMI fails to totally account for the development or progression of RA, researchers speculate that alterations in the production of inflammatory molecules from adipose tissue may help to activate the immune system and slow the process of damage in the joints [14]. Indeed, the release of adipokines from adipose tissue or joint compartments appears to have critical implications in inflammatory and immune responses of rheumatic diseases [15,16]. For instance, in a cohort of nonarthritic individuals with immunoglobulin M rheumatoid factor (IgM RF) and/or anti-citrullinated protein antibody (ACPA) positivity, serum vaspin levels at study entry related to the clinical manifestation of arthritis after a median 22 months of follow-up [17]. No such association was observed with other adipokines (adiponectin, resistin, leptin, chemerin, or omentin). Moreover, no associations were found between adiponectin, resistin or visfatin synovial expression and the development of arthritis [17]. Some researchers have proposed that lower levels of adiponectin in people with obesity are linked with high adiposity (a surrogate for high BMI) and less joint damage in RA [18].

## 2. The Involvement of Adiponectin in Arthritis

### 2.1. Adiponectin in RA 

Adiponectin (also known as Acrp30, AdipoQ and GBP28) has attracted much attention for its potential therapeutic use in metabolic disorders, as this adipokine exerts pleiotropic metabolic effects on insulin sensitivity, inflammation and angiogenesis, primarily via the adiponectin receptors 1 and 2 (AdipoR1 and AdipoR2), as well as the non-signaling binding protein T-cadherin, regulating glucose and lipid metabolism [19,20]. Evidence also indicates that adiponectin serves as a possible link between obesity and cancer [19]. Patients with RA have consistently higher serum [21,22,23] and synovial fluid [24] adiponectin levels than non-RA controls. The data are mixed as to the differential regulation of cytokines by adiponectin: On the one hand, adiponectin is capable of suppressing levels of proinflammatory cytokines tumor necrosis factor alpha (TNF-α) and interleukin 6 (IL-6) that are typically elevated in RA and adiponectin increases levels of the anti-inflammatory cytokine IL-10 in primary human macrophages activated with lipopolysaccharide (LPS) [25]. Conversely, increasing concentrations of adiponectin stimulate cultured synovial fibroblasts from RA and OA patients to produce IL-6 [26]. Adiponectin can also stimulate vascular endothelial growth factor (VEGF) and matrix metalloproteinase (MMP) production in RA fibroblast-like synoviocytes (FLSs), leading to joint inflammation and destruction, respectively [27], and women with erosive OA of the hands have higher serum levels of adiponectin levels compared with those with nonerosive hand OA [28]. Moreover, adiponectin can mediate changes in effector cells in RA disease pathophysiology, inducing gene expression and protein synthesis in human RA synovial fibroblasts (RASFs), lymphocytes, endothelial cells and chondrocytes [29], enhancing prostaglandin E_2_ production in RASFs via AdipoR1 [30,31]. In untreated patients with early RA, serum adiponectin levels have been found to predict radiographic disease progression, independently of metabolic status and potentially confounding factors [32]. Other research has failed to find any association between high serum levels of adiponectin and either homeostasis model assessment for insulin resistance (HOMA-IR) index or common carotid artery intima-media thickness (IMT) measurements [23]. Other investigations into the mechanisms underlying adiponectin function have shown that adiponectin induces production of the proinflammatory cytokine, oncostatin M, in human osteoblasts [33]. 

In severe, infliximab-refractory RA, a negative correlation has been observed between high-grade inflammation (C-reactive protein [CRP]) and low circulating plasma adiponectin levels [34]. That research documented independent, negative correlations between low adiponectin levels with atherogenic dyslipidemia and high plasma glucose levels, findings that are similar to those previously reported in individuals without RA disease, suggesting that low circulating adiponectin levels cluster with features of MetS that are implicated in RA atherogenesis [34]. If higher adiponectin levels are indeed protective against cardiovascular disease and obesity, it may not be wise to modulate those levels. 

### 2.2. Adiponectin in OA 

The somewhat puzzling findings as to the inflammatory activity associated with adiponectin is postulated to be because there are several isoforms that have differing, sometimes counteracting functions [35]. Their selective binding to AdipoR1 and AdipoR2 induces specific intracellular signaling cascades; the oligomerization and expression levels of these receptors determine adiponectin bioactivity [36]. For example, high-molecular-weight adiponectin induces IL-6 in human monocytes but has no effect upon LPS-induced IL-6 secretion, while low-molecular-weight adiponectin reduces LPS-induced IL-6 secretion and induces IL-10 in these cells [36]. Intriguingly, positive correlations have been observed between levels of synovial fluid from OA patients and levels of adiponectin and resistin, whereas conversely, the biological active free form of leptin (not the total leptin) appears to be negatively associated with IL-6 [37]. 

Much higher serum levels of adiponectin, leptin and resistin have been found in patients with severe knee OA compared with controls without radiographic knee OA; that same research also documented weak but positive associations between serum levels of adiponectin, leptin and resistin and synovial inflammation [38]. Another paper, involving female patients with knee OA, identified a significant correlation between synovial adiponectin levels and degradation markers of aggrecan, which suggests that adiponectin regulates the degeneration of cartilage matrix in OA [39]. In an investigation into the effects of adipokines upon the development of OA osteophytes, adiponectin and visfatin stimulated osteoblasts and chondrocytes, respectively, to increase their release of proinflammatory mediators [40]. Adiponectin has been found to enhance nitric oxide, IL-6, MMP-1 and MMP-3 production in OA cartilage and in primary chondrocytes via mitogen-activated protein kinase (MAPK) signaling [29,41]. Similarly, Junker and colleagues found that adiponectin induced p38 MAPK signaling in OA osteoblasts, whereas stimulation with adiponectin, resistin, or visfatin had no effect on Wnt signaling [40]. These findings indicate that adipokines do not directly influence osteophyte development, but that they do influence proinflammatory conditions in OA and that adiponectin possibly mediates cartilage destruction in OA. Other research has reported that monocyte adhesion to the human OA synovial fibroblast (OASF) monolayer is promoted by adiponectin-induced intercellular adhesion molecule 1 (ICAM-1) expression [42]. In contrast, some evidence suggests that serum adiponectin may be protective in OA; a significant, negative association between serum adiponectin and radiographic OA severity in patients with knee OA persisted after adjusting the analyses for age, sex, BMI and duration of disease [43]. 

## 3. Leptin Expression in RA and OA

Leptin, a 16 kDa non-glycosylated protein encoded by the *obese* (*ob*) gene, is mainly secreted by adipose tissue and regulates appetite and obesity by inducing anorexigenic factors and suppressing orexigenic neuropeptides [44]. The release of leptin into the circulation enables it to act peripherally and centrally [45]. After entering the brain via a saturable transport mechanism, leptin’s central location of action is the hypothalamus [45]. This adipokine also has direct effects on non-neural cells [46], as evidenced by its involvement in immunoregulatory functions, as it is capable of inducing T_H_1 immune reactions by increasing the T_H_1 phenotype of the effector CD4 T cell and suppressing the T_H_2 phenotype; leptin can also induce naïve CD4 T cells to proliferate and inhibit memory CD4 T cells from proliferating [47]. 

Increased serum leptin levels have been linked to erosion of cartilage and bone in OA [48], synovitis and cartilage defects, bone marrow lesions and osteophytes [49]. Notably, leptin expression correlates with DNA methylation in OA chondrocytes and leptin’s downregulation dramatically inhibits MMP-13 gene expression [50]. Single nucleotide polymorphism (SNP) analyses have suggested associations between the leptin gene and its receptor gene with OA in both normal weight and overweight Chinese populations [51,52]. Some research has found a significant correlation between leptin messenger RNA (mRNA) expression in advanced human OA cartilage and BMI, suggesting that leptin could serve as a metabolic link between obesity and OA [53]. That same research also found that leptin expression was significantly increased in synovial fluid, indicating that leptin was locally produced as opposed to diffusing from plasma to synovial fluid via the synovial membrane. The ability of leptin to stimulate IL-1β production and increase MMP-9 and MMP-13 protein expression in OA and normal chondrocytes supports the contention that leptin has proinflammatory and catabolic effects in the metabolism of cartilage [53]. Evidence implicates leptin in obesity and joint damage; a significant association has been found between baseline leptin levels and increased biomarkers of bone formation (osteocalcin and PINP) over a 2-year period; conversely, higher levels of soluble leptin receptor (sOB-Rb), which reduces leptin activity, were associated with lower osteocalcin levels at 2 years of follow-up [54]. Interestingly, serum leptin levels are significantly associated with increased knee OA cartilage volume, whereas serum adiponectin levels are significantly associated with lower levels of disease severity in radiographic OA [43]. 

Other research has found that adiposity in leptin-impaired mice does not lead to systemic inflammation and knee OA, which suggests that leptin directly influences knee OA pathogenesis rather than via any correlation with obesity and that the loss of leptin signaling pathways may help to prevent the development of OA [55]. Assessing leptin expression could potentially be used to measure RA and OA disease activity. Higher serum leptin levels are found in RA patients with high disease activity compared with those with low disease activity [22,56] and a small but significantly positive correlation exists between leptin levels and RA activity [57,58]; no such correlation exists between serum adiponectin levels and RA disease activity [58]. This lack of association between adiponectin levels and disease activity was seen in another study involving patients with knee OA, in whom synovial fluid leptin levels and plasma levels of adiponectin, soluble leptin receptor and free leptin were not significantly different across categories of OA severity, although the ratio of synovial fluid to plasma leptin level was significantly lower in advanced OA than in early disease [59]. In contrast, other research has revealed a close association between synovial fluid leptin levels and OA radiographic severity, which is highest in stage IV disease [60]. 

In vitro investigations suggest that leptin increases production of the proinflammatory cytokine IL-8 in RASFs and OASFs by binding to the leptin receptor (OBRI) and activating the Janus kinase 2/signal transducer and activator of transcription 3 (JAK2/STAT3) signaling pathway, which in turn activates the insulin receptor substrate-1/phosphatidylinositol 3 kinase/Akt/nuclear factor-κB (IRS1/PI3K/Akt/NF-κB)-dependent pathway and leads to p300 recruitment [61]. Similarly, leptin induces IL-6 expression in OASFs by activating the OBRl receptor, which in turn activates the IRS-1, PI3K, Akt, and AP-1 signaling pathways and thus upregulates IL-6 expression [62]. Table 1 summarizes the involved receptors, downstream and targeted signaling molecules, and target genes involved in RA and OA. It shows that many leptin receptors exist in individual cells (osteoblasts, FLSs and chondrocytes), but not in the brain. Evidence of brain leptin receptor expression in RA/OA will require much more research. Leptin also increases vascular cell adhesion molecule 1 (VCAM-1) expression in human and murine chondrocytes [63]. Importantly, VCAM-1 functions as a cell adhesion molecule, mediating leukocyte recruitment and extravasation from circulating blood to inflamed joints [63]. Interestingly, leptin appears to induce MAPK signaling in both human chondrocytes [64] and RA FLSs [65]. The presence of leptin stimulates oncostatin M production in osteoblasts from healthy human donors [66]. 

## 4. Resistin in Arthritis

### 4.1. Resistin in RA

Adipocyte-derived expression and secretion of resistin, a small cysteine-rich adipokine, is linked to inflammation and insulin resistance [68,74]. Some researchers have documented positive correlations between serum resistin levels and inflammatory status (erythrocyte sedimentation rate [ESR], CRP) as well as clinical disease activity (28-joint count Disease Activity Score [DAS28]) in patients with RA [22,75]. Significantly higher resistin levels have been observed in synovial sublining layers from RA patients than from OA patients [75]. Šenolt and colleagues have documented resistin expression within several different cell types within the synovial tissue, including synovial fibroblasts, and in different inflammatory cell types found in RA synovium such as macrophages, B lymphocytes and plasma cells [75]. They proposed that resistin is a secreted signaling molecule that helps to activate these cell types in chronic inflammatory states such as RA. Subsequent investigations support this contention, showing positive associations between serum levels of resistin and leptin with CRP levels in RA, indicating that resistin and leptin act as proinflammatory cytokines in this disease [22]. Indeed, resistin has been found to specifically enhance the concentrations of chemokines CXCL8 and CCL2, as well as IL-6, in RA FLSs; transfecting the FLSs with adenylate cyclase-associated protein 1 (CAP1, a receptor for resistin) significantly reduced CXCL8 expression, which implicates the involvement of the resistin-CAP1 pathway in chemokine production in RA synovial tissue [67]. Plasma resistin levels also correlate with coronary artery calcification, a marker of coronary atherosclerosis [74]. 

Interestingly, despite finding evidence in support of resistin as a significant mediator of the inflammatory process in RA, Yoshino and colleagues (2011) found that serum resistin levels did not differ between RA patients and healthy controls [22], which is backed by other investigations [76,77]. In contrast, one small study found higher serum and synovial resistin levels in RA patients compared with OA patients, supporting a role for resistin in autoimmune inflammatory rheumatologic disease [78]. The study evidence also suggested that high synovial fluid resistin levels may be a poor prognostic factor for RA in terms of disease progression and radiologic joint damage. 

Other researchers have described how resistin directly induces significant increases in VEGF expression in endothelial progenitor cells (EPCs) and promotes EPC homing into the synovium, inducing RA angiogenesis; inhibiting resistin reduces EPC homing into synovial fluid and angiogenesis in mice with collagen-induced arthritis [68]. Those researchers detail the involvement of the protein kinase C delta (PKC-δ) pathway in resistin-induced EPC migration and tube formation; this investigation was the first to show that resistin induces EPC migration and tube formation by downregulating microRNA 206 (miR-206) expression via the PKC-δ/AMPK (AMP-activated protein kinase) signaling pathway, which involves VEGF expression in primary EPCs. This clarification of the mechanisms underlying RA pathogenesis highlights resistin as a therapeutic target in RA and is confirmed by an investigation into resistin gene expression in pathogenetic leukocyte subsets from patients with active RA treated with TNF-α inhibitor therapy (adalimumab) for 3 months [79]. Among those who responded to adalimumab, resistin (*RETN*) gene expression was significantly downregulated in CD14^+^ and CD4^+^ monocytes, but was unchanged in CD8^+^ T cytotoxic lymphocytes and CD19^+^ B lymphocytes [79]. Conversely, *RETN* gene expression increased in a patient who failed to respond to adalimumab. Some research has explored whether selected gene polymorphisms in Chinese Han patients with RA and healthy controls are associated with RA susceptibility and clinicopathological characteristics [80]. The analysis examined four *RETN* single nucleotide polymorphisms (SNPs rs3745367, rs7408174, rs1862513, and rs3219175). Those carrying the C allele of the *RETN* SNP rs7408174 and those with the AG allele or who had at least one A allele of the SNP rs3219175 were more likely than wild-type carriers to develop RA. In addition, RA patients carrying the AG allele of the *RETN* SNP rs3219175 had higher serum CRP levels compared with controls, and had a high likelihood of being prescribed TNF inhibitors. Besides the risk for RA, genetic variation in *RETN* is linked to a higher likelihood of other diseases, such as MetS and colon cancer, while the *RETN* SNP rs186513 is implicated in a higher risk of type 2 diabetes [74]. Moreover, *RETN* SNPs correlated with lung cancer progression in patients with Chinese Han ethnicity [74]. 

### 4.2. Resistin in OA

Resistin may possibly serve as a drug target in OA: Positive correlations have been observed between resistin and inflammatory factors (IL-6, MMP-1 and MMP-3) in human OASFs [81]. Moreover, the researchers found a correlation between release of resistin from cultured OA cartilage and resistin levels in synovial fluid. Similarly, one study has reported that levels of circulating leptin resistin, IL-6 and IL-17, were positively correlated with clinical disease activity in Mexican patients with RA [82]. 

## 5. Visfatin in Arthritis

### 5.1. Visfatin in RA 

Visfatin (otherwise known as pre-B-cell colony-enhancing factor [PBEF] or nicotinamide phosphoribosyltransferase [NAMPT]) is actively involved in the synthesis of cellular nicotinamide adenine dinucleotide (NAD^+^) and helps to regulate cellular growth, angiogenesis and apoptosis in mammalian cells [83]. Visfatin also triggers the release of cytokines, chemokines and proinflammatory enzymes that are characteristically present in RA joints [84] and is overexpressed in plasma and synovial fluid of several inflammatory diseases, including RA and OA [85,86], suggesting that visfatin promotes their development. This is supported by findings showing that visfatin/PBEF contributes to proinflammatory chemokine production in RASFs, matrix-degrading factors and pro-angiogenic molecules in RA synovial tissue [69]. Moreover, other researchers have demonstrated a positive correlation between circulating visfatin levels and RA disease activity as assessed by DAS28 and CRP levels [87]. Interestingly, although the study also reported significantly higher circulating adiponectin levels in RA patients than in controls, there was no apparent link between circulating adiponectin and disease activity.

Insulin-like growth factor-1 (IGF-1) is implicated in the synthesis and repair of cartilage matrix. Visfatin inhibits IGF-1 function in chondrocytes by prolonging the activation of the extracellular signal-regulated kinase (ERK)/MAPK signaling pathway, independently of IGF-1 receptor activation [70]. Visfatin also inhibits IGF-1-stimulated proteoglycan (PG) synthesis, basal and IGF-1-stimulated collagen type II expression and synthesis [70]. These findings help to clarify the local effects of visfatin on joint tissue and its effects upon inflammatory disease.

Substantial epidemiological data characterize cigarette smoking as an important risk factor for RA and attest to the negative impacts of smoking upon all stages of RA disease [88]. Cigarette smoking reduces the clinical response to antirheumatic therapy and to treatment with TNF inhibitors in particular, especially infliximab [88]. In preclinical and early-stage disease, evidence suggests that smoking may interact with HLA-DR shared epitope genes and encourage the development of anticitrulline antibody-positive RA [89]. A strong, positive association has been observed between smoking and radiographic progression in early RA [88]. In established RA disease, cigarette smoking has been associated with progressive joint damage, persistently active RA and the development of rheumatic nodules [89]. Smoking is also associated with high concentrations of inflammatory cytokines [88] and inversely associated with circulating levels of IGF-1 [89]. Intriguingly, researchers have demonstrated lower serum levels of leptin and adiponectin in smokers than in non-smokers, whereas smoking appears to have no such effect upon resistin and visfatin levels, which are similar between smokers and non-smokers [89]. 

Visfatin is known to increase cardiovascular risk. In untreated patients with early-stage RA, significant positive concentrations have been observed between visfatin and biochemical markers of severe metabolic disturbance (insulin and insulin resistance, total and LDL cholesterol and triglycerides), although other studies have failed to find an association between visfatin concentrations and coronary artery calcification scores in RA, between visfatin concentrations and carotid artery IMT, or any relationship between NAMPT polymorphisms, disease susceptibility and cardiovascular risk in RA [90]. In patients with established, treated RA, visfatin concentrations have been found to be independently associated with increased diastolic blood pressure and diabetes [90]. Moreover, visfatin concentrations were directly associated with levels of MMP-2 (a plaque stability mediator), even after adjusting for adiposity and Clinical Disease Activity Index (CDAI) scores. It is thought that elevated MMP-2 expression in RA might help to compensate for the visfatin-induced enhancement of cardiovascular risk [90]. Interestingly, several different types of cancers (i.e., colorectal, gastric, breast, prostatic, pancreas and esophageal) exhibit overexpression of visfatin [83]. 

### 5.2. Visfatin in OA

Investigations have confirmed an essential role for visfatin in the destruction of OA cartilage mediated by hypoxia-inducible factor 2-alpha (HIF-2α) [72]. Not only does HIF-2α directly target the *Nampt* gene in articular chondrocytes and OA cartilage, but also, visfatin upregulates mRNA levels and activities of MMP-3, MMP-12 and MMP-13 and downregulates aggrecan expression in chondrocytes, all of which are critical for OA pathogenesis. Inhibiting visfatin enzymatic activity blocks the destruction of OA cartilage [72]. In in vitro investigations, visfatin-induced promotion of IL-6 and TNF-α in human synovial fibroblasts occurs through the ERK, p38, and JNK signaling pathways [71].

## 6. Lipocalin-2 in RA and OA

Lipocalin-2 is upregulated in adipose tissue of obese animals and in vitro evidence suggests that lipocalin-2 homeostatically regulates inflammatory activity and inflammation-mediated adipocyte dysfunction in an autocrine or paracrine fashion [91]. This is supported by findings showing elevated levels of fecal lipocalin-2 in mice with collagen-induced arthritis and concomitant experimental colitis; induction of colitis delayed the onset of arthritis and reduced its severity as compared with the arthritis-only group [92]. Interestingly, the development of arthritis was not affected by colitis severity. Similarly, other researchers have reported that although higher serum lipocalin-2 levels can be used as an indicator of structural damage such as erosions in early-stage RA, they cannot be used to monitor disease activity [93]. A recent investigation into the expression and role of lipocalin-2 in OA osteoblasts and chondrocytes in osteochondral junctions has revealed its importance as a catabolic adipokine and its regulation in osteoblasts by inflammatory, catabolic, and anabolic factors [94]. That investigation also revealed that osteoblasts induced the paracrine expression of lipocalin-2. According to these findings, lipocalin-2 is apparently an active catabolic agent in OA joints and may serve as a link among obesity, aging and OA joint alterations.

## 7. Apelin in RA and OA

Early in vitro investigations into the role of apelin at any of the following concentrations of 0.5, 1, 10, or 100 nM in cartilage metabolism indicated that this cytokine stimulates chondrocyte proliferation and increases MMP-1, MMP-3 and MMP-9 transcript levels, as well as IL-1β protein expression [73]. These results were supported by in vivo findings: After rats were administered intra-articular injections of apelin (1 nM), MMP-1, MMP-3 and MMP-9 were upregulated, ADAMTS-4 and ADAMTS-5 mRNA levels were markedly increased, as were IL-1β levels, while levels of collagen II gene and protein expression were reduced. Moreover, proteoglycan was depleted in articular cartilage after apelin treatment. In patients with RA, research has reported finding a strong inverse association between apelin concentrations and those of MMP-9 [95]. Patients with early RA exhibit significantly lower serum apelin levels compared with apelin profiles of healthy controls [96]. The published evidence suggests that it could be worth investigating drugs that specifically target apelin and thus inhibit the development of arthritic diseases such as RA and OA.

## 8. Omentin, Vaspin and Nesfatin in RA, OA, and Other Arthritic Diseases

Similarly, another anti-inflammatory adipokine, omentin, is associated with lower levels of MMP-3 in RA [97], while nesfatin-1 has been found to be inversely associated with carotid IMT (80), suggesting that certain adipokines may protect against cardiovascular disease in RA. Patients with psoriatic arthritis exhibit higher serum levels of omentin and leptin, but lower levels of adiponectin and chemerin, compared with healthy controls [98]. Interestingly, whereas higher serum levels of omentin and leptin are positively correlated with numbers of osteoclast precursors in peripheral blood, lower serum adiponectin levels in psoriatic arthritis are negatively correlated with osteoclast precursors [98]. Those researchers also described finding a positive correlation between leptin and Psoriatic Arthritis Joint Activity Index scores. Serum levels of omentin are also significantly higher in patients with juvenile idiopathic arthritis (JIA) compared with healthy controls; moreover, omentin serum levels are higher in JIA with active joints compared with JIA without active joints, and a positive significant correlation has been observed between omentin serum levels and the presence of active joints in JIA [99]. That same investigation failed to find any such associations between these parameters of disease activity and serum levels of vaspin in JIA [99]. Omentin apparently has no effects upon central effector cells in RA pathophysiology, despite its presence in the synovium and synovial fluid from RA and OA patients [100]. Interestingly, not only do synovial fluid levels of omentin and vaspin appear to differ at the site of local inflammation in patients with RA and OA, but also, patients with RA have demonstrated lower levels of omentin and higher levels of vaspin in synovial fluid compared with patients with OA [101]. Those researchers found that synovial fluid levels of vaspin, but not of omentin, tended to correlated with DAS28 scores, but neither adipokine was correlated with serum CRP or synovial fluid leucocyte counts [101]. Although omentin seems to have anti-inflammatory and antiatherogenic properties in obesity and displays negative associations in inflammatory bowel disease and MetS, these effects may not occur in RA and OA [100]. It may be worth targeting nesfatin-1 in OA; elevated levels in serum and synovial fluid from patients with knee OA have been found to be significantly associated with disease severity as determined by Kellgren–Lawrence grading criteria [102]. Moreover, it is speculated that nesfatin-1 may contribute to pathophysiological changes in OA [103]. Nesfatin-1 has been found in articular cartilage in patients with knee OA, who exhibit significantly higher serum levels of nesfatin-1 compared with serum from healthy controls [103]. Furthermore, serum nesfatin-1 levels are significantly correlated with high-sensitivity CRP levels, while synovial nesfatin-1 is significantly correlated with IL-18 levels in patients with OA [103]. 

## 9. Other Adipokines

Importantly, besides those adipokines covered in this review, we cannot discuss other more recently discovered adipokines including adipolin, acylation-stimulating protein, fasting-induced adipose factor, retinol-binding protein-4, and serum amyloid A3, because no available data provide evidence for their roles in RA and OA disease. Interestingly, related research has found that the novel adipokine fatty acid-binding protein 4 (FABP4), closely associated with obesity and metabolic diseases, is significantly higher in the serum and synovial fluid of patients with RA than in those of OA patients [104], while plasma and synovial fluid levels of FABP4 are significantly higher in OA patients than in those of healthy non-OA controls [105]. Furthermore, recent experimental research has indicated that in FABP4 knockout mice (KO) with obesity induced by a high-fat diet, cartilage degradation is significantly alleviated after 6 months of daily oral gavage with a selective FABP4 inhibitor has suggested that FABP4 may be a potential therapeutic target in OA [106]. Further explorations into the pathogenic aspects of novel adipokines involved in obesity may well uncover other such links into RA and OA disease activity; the space limitations of this review prevent us from researching this aspect.

## 10. Summary and Future Directions

**Figure 2:**
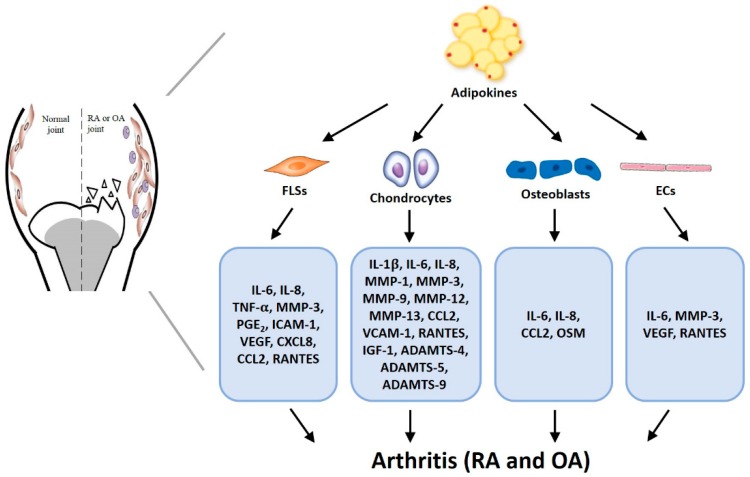
Critical pathways involving adipokines in arthritic diseases. Adipose tissue paracrine signaling in RA and OA demonstrates systemic links between adipokines and arthritic disease.

Controversially, obesity does not necessarily influence the likelihood of developing RA; the evidential link is more definite for OA, where leptin appears to be a metabolic link between obesity and OA. Other adipokines, such as visfatin and resistin, also play important roles in arthritis pathogenesis (Table 1 and Figure 2). In agreement with previous summaries of evidence on the interaction of adipokines with RA and OA [107,108], the evidence discussed in this review suggests that it could be useful to develop therapeutic molecules that target individual adipokines. We have found no published evidence for any natural product that inhibits adipokines and potentially treats arthritis. To help overcome the lack of treatment opportunities, our laboratory is constructing a full-length adipokine containing luciferase for use as a screening model to identify and test natural products or pharmacochemical structures able to target specific adipokines involved in RA and OA disease. We are also working on the design of anti-adipokine antibodies that we will test for their ability to detect the early development of arthritic diseases, so that in future anti-arthritic therapy can be administered early to prevent disease progression. 

## Figures and Tables

**Table 1:** Adipokines implicated in rheumatoid arthritis and osteoarthritis.

Stimulation	Target Factors		Effects in Tissue	Disease	Receptor	Known Pathways	References
***Adiponectin***							
	IL-6	↑	FLSs	OA & RA	AdipoR1	AMPK/p38/IKKαβ and NF-κB	[26]
	VEGF, MMPs	↑	FLSs	RA	N/A	N/A	[27]
	IL-6, RANTES, MMP-3	↑	FLSs, lymphocytes, endothelial cells, and chondrocytes	RA	N/A	PKA/NF-κB/p38MAPK/PKC	[29]
	PGE2	↑	FLSs	RA	AdipoR1	NF-κB	[30,31]
	OSM	↑	Osteoblasts	RA	N/A	PI3K/Akt and NF-κB	[33]
	IL-6, IL-8, and CCL2	↑	Osteoblasts and chondrocytes	OA	N/A	p38/MAPK	[40]
	IL-6, MMP-1,-3	↑	Chondrocytes	OA	N/A	p38/ERK1/2/JNK	[41]
	ICAM-1	↑	FLSs	OA	AdipoR1	LKB1, CaMKII, AMPK, and AP-1	[42]
	VCAM-1	↑	Chondrocytes	RA & OA	N/A	JAK2 and PI3K	[63]
***Leptin***							
	MMP-13	↑	Chondrocytes	OA	N/A		[50]
	IL-1β, MMP-9 and MMP-13	↑	Chondrocytes	OA	OBRb		[53]
	IL-8	↑	FLSs	RA & OA	OBRI	JAK2/STAT3 and IRS1/PI3K/Akt/NF-κB	[61]
	IL-6	↑	FLSs	OA	OBRI	IRS-1/PI3K/Akt, and AP-1	[62]
	VCAM-1	↑	Chondrocytes	RA & OA	N/A	JAK2 and PI3K	[63]
	ADAMTS-4, -5 and -9	↑	Chondrocytes	OA	N/A	MAPK and NF-κB	[64]
	IL-6	↑	FLSs	RA	OBRb	JAK2/STAT3	[65]
	OSM	↑	Osteoblasts	RA	OBRI	AKT/miR-93	[66]
***Resistin***							
	CXCL8, CCL2 and IL-6	↑	FLSs	RA	N/A	CAP1	[67]
	VEGF	↑	EPCs	RA	N/A	PKC-δ/AMPK/miR-206	[68]
***Visfatin***							
	IL-6 and IL-8, CCL2 and MMP-3	↑	FLSs	RA	N/A	p38 pathway	[69]
	IGF-1	↓	Chondrocytes	OA	IGF-1R	ERK/MAPK signaling pathway	[70]
	IL-6 and TNF-α	↑	FLSs	OA	N/A	ERK/p38/JNK and miR-199a-5p	[71]
	MMP-3, -12, and -13	↑	Chondrocytes	OA	N/A	HIF-2a	[72]
***Other adipokines***							
	MMP-1, -3 and -9, ADAMTS-4 and -5, IL-1β		Chondrocytes	OA	N/A	JNK, ERK and MAPK	[73]

IL-6, interleukin 6; FLSs, fibroblast-like synoviocytes; OA, osteoarthritis; RA, rheumatoid arthritis; AdiopoR1, adiponectin receptor 1; AMPK, AMP-activated protein kinase; IKKα/β, IκB kinase alpha/beta; NF-κB, nuclear factor-kappa B; VEGF, vascular endothelial growth factor; MMP, matrix metalloproteinase; RANTES, regulated upon activation, normal T cells expressed and secreted; PKA, protein kinase A; p38MAPK, P38 mitogen-activated protein kinase; PKC, protein kinase C; PGE2, prostaglandin E2; OSM, oncostatin M; PI3K, phosphatidylinositol 3 kinase; IL-8, interleukin-8; CCL2, chemokine (C-C motif) ligand 2; ERK1/2, extracellular signal-regulated protein kinases 1 and 2; JNK, c-Jun N-terminal kinase; ICAM-1, intercellular adhesion molecule 1; LKB1, liver kinase B1; CaMKII, Ca^2+^/calmodulin-dependent protein kinase II; AP-1, activator protein 1; VCAM-1, vascular cell adhesion molecule 1; JAK2, Janus kinase 2; IL-1β, interleukin-1 beta; OBRb and OBRl, long isoform of leptin receptor; STAT3, signal transducer and activator of transcription 3; IRS1, insulin receptor substrate-1; ADAMTS-4, ADAM metallopeptidase with thrombospondin type 1 motif 4; ADAMTS-5, ADAM metallopeptidase with thrombospondin type 1 motif 5; ADAMTS-9, ADAM metallopeptidase with thrombospondin type 1 motif 9; miR, microRNA; CXCL8, C-X-C motif chemokine ligand 8; CAP1, adenylate cyclase-associated protein 1; EPCs, endothelial progenitor cells; IGF-1, insulin-like growth factor 1; IGF-1R, IGF-1 receptor; TNF-α, tumor necrosis factor alpha; HIF-2α, hypoxia-inducible factor 2 alpha.
